# The Foreign Relations Committee of the National Association of Dental Faculties

**Published:** 1899-11

**Authors:** 


### COMMUNICATIONS.

### The Foreign Relations Committee of the National Association of Dental Faculties.

*To Whom It May Concern:*—American dental colleges have in the past encountered great perplexities in properly grading students from foreign, non-English-speaking countries. It was quite impossible for those unacquainted with the mother tongue of proposed matriculants to determine their educational qualifications, or their fitness for reception as first year students.

The same embarrassment existed in ascertaining who were entitled to advanced standing, and to reception into the junior and senior classes. Institutions for the professional instruction of dentists are of but recent origin, the first having been established in America. In most foreign countries, until within a very few years, while professional schools taught medical science very thoroughly, dental education was confined to a comparatively few lectures in universities and hospitals, the practical knowledge being imparted by private schools and preceptors.

Our difficulty consisted in the determination of what credit to allow foreign students for such courses. Certain unprofessional men in foreign lands, who were acting as private preceptors, and who desired to assist a protege, sometimes assumed for the occasion academic airs, and granted certificates of study for use in America. These, prepared in a foreign tongue, were presented to an American dental college as evidences of regular courses taken in dental study. If a translation was required, that aided the college authorities but little, as the curriculum was probably incorrectly stated.

There was no list of properly equipped foreign dental schools obtainable, and so it was utterly impossible to determine what the certificates presented were worth. In some instances formidable looking documents, with great seal attached, were subsequently found to be only the certificates of some immigration or other bureau. These were presented with a false statement of their import, and if refused by one dean were taken elsewhere, until a school was found which would accept them. Under these conditions, with no common understanding among American colleges, illiterate and uninstructed men, in some instances servants of foreign dentists who owed American schools little of good will, were fitted out with certificates which secured for them admission to the senior class of some American college, and they returned home with an American diploma at the end of a single term.

So often had reputable American dental schools thus been imposed upon, that some remedy for this state of affairs became imperative. Accordingly, the National Association of Dental Colleges, which had been duly organized, and which included in its membership all those which had professional recognition in this country, at its annual meeting for 1897 appointed a Foreign Relations Committee, which was subsequently charged with the regulation and control of all American dental educational matters connected with foreign countries, and with the suppression of all irregular and fraudulent American diploma-granting institutions.

This committee, in the furtherance of its work, has appointed in most foreign countries from which students come to attend American dental schools, an advisory board, to which the credentials of all foreign students in American dental colleges must be referred. The National Association has decreed that no American college having its endorsement for reputability shall allow advanced standing upon certificates, or other evidences of study, that have not been verified and approved by the advisory board for the country from which the proposed student comes. All applications made in America for admission of foreign students to the first year's course of our schools, must also be referred by them to the Foreign Relations Committee for like information and approval. This action is imperative, and will henceforth be enforced upon all reputable American dental schools.

It seems proper that the Foreign Relations Committee, with which the advisory boards are to co-operate, should indicate what is to be expected of them, and suggest methods of procedure. While it will be quite competent for the boards to organize and draw up rules for their own internal government, in the interest of harmony and effectiveness, it appears wise that some expression be given by the Foreign Relations Committee as to what is advisable.

There is danger that improper precedents may be established if any precipitate action is taken by individual members of the foreign boards. No American college can henceforth accept a foreign student until he has the approval of the board appointed for the country from which he comes, subject always to appeal to the Foreign Relations Committee. Hence full conference and mutual understanding is urged before decisive measures are adopted, and great caution in the certification of students to American schools is recommended. For the present, at least, it is urged that no individual member of a foreign board attest with his signature of approval the credentials of any foreign student until after full consultation with his associates of the same board.

In determining preliminary educational qualifications, the foreign equivalent for those of the National Association of Dental Faculties must be ascertained, and no student should be certified who does not positively demonstrate to the entire satisfaction of the members of the advisory board that he unquestionably possesses them.

No student should be certified who has not a sufficient knowledge of the English language to comprehend college lectures, or if he is without this, it should be explicitly so stated in the certificate. These requirements are necessary for acceptance as a first term student.

To obtain advanced standing in an American school, something more is necessary. Aside from the preliminary requirements, the student, if he would be admitted to our junior or senior classes, must have taken one or more terms in a foreign dental school which shall have been previously accepted by the Foreign Relations Committee as maintaining a proper standard, not only as to preliminary qualifications, but in practical instruction as well. If foreign governments are justified in refusing to accept and register graduates of American colleges because of imperfect literary acquirements, we are equally warranted in declining to accept and give credit for courses that are below our standard in practical instruction. Surely, in the equipment of a practicing dentist the latter is as important as the former, and the foreign school which does not include in its curriculum the practical instruction given in American colleges can not expect to receive full credit for terms spent in study by its students.

It is expected, then, that applicants will not be certified for advanced standing in American schools by the members of advisory boards, unless the colleges from which they come have been approved by the Foreign Relations Committee as maintaining a standard in practical teaching that can be accepted as a full equivalent for ours. It is incumbent upon us to sustain the character of our diplomas, and to guard against the reception of certificates from those which are inferior.

It is unfortunately true that there have been in the past institution in America which were engaged in the selling of dental and other diplomas in foreign countries. Nearly all of these have had their headquarters in the State of Illinois. For about twenty-five years there has been a law peculiar to that State, enacted for the purpose of promoting business enterprises, and excellent in its character when confined to legitimate objects, but which was so loosely drawn that under it there existed the possibility of obtaining charters for so-called dental, medical and other colleges with pretentious names and titles, which were practically beyond the control of the educational laws of the State.

These fraudulent institutions possessed actual charters from the State, which were owned and controlled, usually by some man who was not legitimately a member of any profession. For a long time their work was confined to foreign countries, and so secretly done that public attention in America was not attracted to them. Of course these institutions had no actual existence, and no responsible head which could be located.

At last they became so bold that they openly advertised their wares in this country, and the educational and other boards of the State of Illinois attempted their suppression, but failed. The publication of the report of the Foreign Relations Committee of the National Association of Dental Faculties, a little more than a year ago, extracts from which were scattered broadcast and placed in the hands of every member of the State Legislature, aroused such a storm of indignation that the legal counsel of the committee was enabled, with the help of other interested persons, to obtain the passage of a law authorizing the authorities, on the presentation of the proper proofs, to secure the annulment of the fraudulent charters. It was found impracticable to repeal the law under which the charters were obtained, but it was so modified as to prevent the issue of further franchises of that nature.

To exterminate those already in existence, it is necessary to proceed against each separately for the annulment of the charter which it possesses. Proof must be secured of the issuing of a diploma for improper considerations and without the usual attendance. The Foreign Relations Committee, acting for the National Association of Dental Faculties, has already begun proceedings against such as it has proof to condemn, and it asks the advisory boards to assist in wiping out this disgrace to the American professional name by securing the evidence upon which these harpies may be convicted. It must be remembered that these proofs almost exclusively exist in foreign countries.

It is earnestly requested that the members of foreign advisory boards furnish to the Foreign Relations Committee a list of all schools that give dental instruction in the countries which they represent, with a full description of the course of study pursued in each, especially in the practical branches, the time devoted to each individual study, and the character of the practical instruction given, with any other information that will enable the Foreign Relations Committee properly to grade such schools, and to give due credit for any full equivalents of courses in American dental colleges.

A list of the members of the National Association of Dental Colleges to date is appended. No school is recognized by the Association unless it has such membership, nor will certificates from outside American dental schools be received. It seems equally proper that no certificate from any foreign school that is not recognized by the Association as maintaining a proper standard should entitle a student to advanced standing. Such recognition will be extended by the Foreign Relations Committee, and recommended to the Association as fast as the character of the instruction given in such school and its claims to be accepted as a full equivalent for a course in an American dental college can be determined.

A list of the foreign countries represented, or to be represented, by advisory boards, is also added, with the appointments so far as made. It is earnestly requested of members of foreign advisory boards that full data as to the college from which each appointee or nominee graduated, with the year of graduation, the qualifications for practice in the country which each represents, with full name and address, be given in every instance in which it is left blank in this circular. Such information should be addressed to the Chairman of the Foreign Relations Committee.

Buffalo, N. Y., U. S. A., Nov. 1, 1899.

William C. Barrett, *Chairman*,

208 Franklin St., Buffalo, N. Y.

Simeon H. Guilford,

1728 Chestnut St., Philadelphia, Pa.

John D. Patterson,

9th and Walnut Sts., Kansas City, Mo.

Truman W. Brophy,

126 State St., Chicago, Ills.

Henry W. Morgan,

211 N. High St., Nashville, Tenn.

*Foreign Relations Committee.*

